# Doctors and Patients

**Published:** 1887-11-26

**Authors:** 


### The Doctor's Pulpit.

Doctors and Patients.

The case of the German Crown Prince has given rise to certain incidents which do not quite coincide with very precise notions of professional etiquette. Similar departures from the strict code of conventional propriety in the case of highly-placed persons may be recalled by the memories of those who have had even a moderately lengthened experience of medical life. The journals devoted to medicine have not usually thought it desirable to mark those breaches of etiquette, wisely considering that as the "life is more than meat," so it is more than rules of conventional propriety, which, in ultimate analysis, are merely rules of convenience.

It would not be interesting to a large section of our readers to discuss that branch of professional ethics which regulates the behaviour of medical men among themselves. But it may be interesting, and it is certainly important, for all people sometimes to consider the rules of conduct which the doctor should observe towards his patient, and those by which the patient also should regulate his behaviour towards the doctor. The medical man holds an absolutely unique relation to his patient—a relation not merely different from that of the shopkeeper, but also different entirely from that of the lawyer, and even the clergyman. This is true of all classes of medical men, consultants, specialists, and general practitioners. But most of all is it true of him who is in the truest sense "the doctor"—the family medical attendant.

In medical practice there are two standpoints from which to view every question—the doctor's and the patient's. According as a given circumstance is looked at from one or other of these will that particular circumstance be judged. It need not be said that he who desires to act rightly will always endeavour to look at things from both standpoints.

The doctor naturally enough regards the patient from his own point of view. To him patients are good, middling, and bad, according as they possess certain well-known characteristics. A typically good patient, for example, is Mr. Smyles. He is an old gentleman of abundant means and amiable temper, who likes to be visited professionally at least three times a week. He is always pleased with his physic; never questions the doctor's judgment in any matter great or small; never desires a "consultation," being persuaded that Doctor Gentian is the cleverest fellow in the world. At Christmas and at Midsummer a seventy guinea cheque is sent in with Mr. Smyles' very sincere compliments. From the doctor's standpoint this gentleman is absolutely faultless, and only is not canonised because he is not dead.

A very different type of person is Mrs. McSnapper. She never ails anything, though she is always ill. When she sends for the doctor she says to her servant, "Now be sure you see Dr. Gentian himself, and tell him to come at once. He need not send Mr. Second Fiddle, because I won't have him." The medicine prescribed never does Mrs. McSnapper any good; she is sure the chemist made a mistake in mixing it. Her digestion is bad, and she can't sleep. She has not slept a wink for three nights. Her husband is neither kind nor sympathetic; her servants are lazy, and won't make her a cup of tea at three o'clock in the morning without grumbling so much that she cannot enjoy the tea when it comes. When the doctor's bill is sent in she cannot for her life understand how it is so large. She has never been ill—not to say really ill. She is sure he must have raised his fees, or his bookkeeper has made a mistake. She will keep an account of all his visits next year and see for herself how much he charges. It shall be paid this time, but if it be so big again she will send for Mr. Smallfee, who lives at the corner and doesn't pretend to keep up so much style as Dr. Gentian. Mr. Smallfee used to be a chemist, and has some regard for the pockets of his patients, having known what it was to want a five-pound note himself. It need not be said that Mrs. McSnapper is not a model patient from the doctor's point of view. In the very inmost and profoundest depths of his consciousness he regards her as a nuisance—it is to be feared that he whispers to himself a "confounded nuisance," and if it were not for his half-dozen little boys and girls he would give himself the savage pleasure of telling Mrs. McSnapper to go and drown herself in a big physic bottle.

This good lady, however, is not placed in the doctor's black list. She pays her bills with moderate regularity, or at least her husband does for her, and that is one of the most saving of all graces in the doctor's code of virtues. Mrs. McSnapper may need the fires of purgatory before she can worthily be placed on his list of *bonâ-fide* saints; but so long as she continues to pay her bills he will not condemn her absolutely to outer darkness.

The only really hopeless and for-ever-to-be-execrated person is Mrs. Languid. She never has the doctor out of her house all the year round. She wants the newest physic, the costliest liniments, and the most elegant pills in the surgery. Every few months she thinks she should see a "physician," either for herself or her children. She pays the physician his eight or ten guinea fee, and never complains. She worries the doctor's life out with her vagaries and whims; but when Christmas comes round, the account is so large she dare not show it to her husband, and so it is locked away in her cabinet, where it remains until the next Christmas arrives. Ah, Mrs. Languid! If you could but see sometimes what is passing inside Dr. Gentian's brain when he is looking so pleasant and patient as you make your querulous complaints to him, you would bid him good morning very promptly, and ask the housemaid to lock the door after him, lest he should return and say something to you that would make you spring "ceiling high," as Carlyle says, to come down again with your body pounded to a jelly and some new ideas in your mind.

We will frankly confess the truth. The family doctor is the most patient, forbearing, and forgiving of men. Having known by experience what his life is, and having ceased to pursue that line of practice, we feel that we may, without fear of being charged with pleading our own cause, say what is in our heart. It can do nothing but good to patients to know what the doctor sometimes feels, and he, we are sure, will forgive us. It is probable that no class of men give so much of all that is best in human nature to their daily work as doctors. They make up their minds very early in their career that duty calls them to be at everybody's service at every hour, both of day and night. They attend calls from all kinds of persons, often not knowing whether they will ever receive any reward or not, and not seldom being quite sure that not a single penny, or even a word of thanks, will be vouchsafed to them. They take upon them responsibilities which are nothing less than terrible, and which they, accustomed as they are to such responsibilities, feel to be terrible. They run risks to themselves and their children which often daunt their hearts and drive them almost to desperation. No class of men deserve better of their fellows than family doctors, and we are glad to think and know that the majority of their patients value them according to their worth, and look upon them, not only as sober heroes in life's great conflict, but as trusted, loved, and honoured friends. "Out of the abundance of the heart the mouth speaketh," and we have written at so much at length on the patient from the doctor's point of view, that we have left no space for the consideration of the question from the patient's standpoint. That must form the subject of another sermon.

